# Green Extraction and Valorization of By-Products from Food Processing

**DOI:** 10.3390/foods13101589

**Published:** 2024-05-20

**Authors:** Selin Şahin, Ebru Kurtulbaş

**Affiliations:** Chemical Engineering Department, Faculty of Engineering, Istanbul University-Cerrahpaşa, 34320 Istanbul, Turkey; ebru.kurtulbas@iuc.edu.tr

## 1. Introduction

Agro-industrial valorization has been a hot topic recently since it leads to resource conservation and is economically and environmentally valuable [1,2,3,4,5,6]. Taking into consideration the remarkable percentage of waste added by the food industry, a biorefinery approach should be adopted by using this waste as a raw material and converting it into biogas, biofuel, and bioactive substances, considering how rich they are in terms of high value-added products [7,8,9,10,11,12]. Within the scope of the current Special Issue, our focus was placed on the biomaterial recovery of the by-products from food processing. However, this process requires a sustainable solution. Therefore, our main goal should be to examine green extraction methods, which can be used as one of the sustainable solutions.

Green extraction techniques play a crucial role in this process by utilizing environmentally friendly methods to extract bioactive compounds from relevant by-products [13,14,15]. These techniques involve the use of low-environmental-impact technologies and green solvents to extract antioxidants and other valuable components from food processing waste [16]. By focusing on sustainability and process efficiency, these methods aim to maximize the utilization of biowaste while minimizing their environmental impact [17,18,19]. These methods offer sustainable alternatives to traditional extraction processes, enabling the production of functional foods, cosmetics, and other value-added products from waste materials [20]. By adopting innovative and eco-friendly extraction processes, the food industry can not only reduce waste but also enhance the economic viability of by-product valorization initiatives [21].

Consequently, the valorization of by-products from food processing through green extraction techniques represents a promising avenue for promoting sustainability, reducing waste, and creating value from underutilized resources. By leveraging environmentally friendly extraction methods and focusing on the efficient recovery of bioactive compounds, the food industry can contribute to a more circular economy and sustainable food production practices [22]. Embracing green extraction technologies not only aligns with the principles of sustainable development but also offers opportunities for innovation and the development of novel products with enhanced nutritional and functional properties.

## 2. An Overview of Published Articles

Five research articles within the scope of the present Special Issue were published. Various biowaste (fish by-product, grapefruit peel, black raspberry pomace, spent coffee grounds, and sour cherry pomace) created from the food industry were examined as sources of bioactive substances.

Wang et al. (Contribution 1) evaluated fish soup processing waste, a by-product stream that has not been researched extensively [23]. Even though waste by-products from aquatic product processing (skins, bones, and scales) form a large amount (around 30–70%) of the total output, they are not utilized properly, especially when looking at their considerable environmental management expenses. Therefore, the research group investigated the enrichment of crucian carp soap with fish by-products. In a related study, comminution combined with pressure conduction was used to develop a new product by examining the impact of mixing ratios of crucian carp original soup (OS) and residue soup (RS) on the flavor and nutrient composition of the products. The optimal combination (7/3, OS/RS) with the best product in terms of nutritional and flavor properties was also identified.

Öztürk et al. (Contribution 2) have drawn attention to the main waste by-product of the juice industry [24]. Citrus peel accounts for almost 50% of this type of fruit’s total weight. Moreover, this biowaste is also rich in pectin. So, grapefruit peels were used as a source of pectin in the related study. Microwave-assisted high-pressure CO_2_/H_2_O (MW-HPCO_2_) system, as an advanced green extraction technology, was utilized to extract pectin from the peels. The system’s conditions (time, temperature, and solvent to solid ratio) were optimized through a Box–Behnken design of the response surface method (RSM). The proposed method (147 °C of treatment temperature, 3 min of extraction time, and 1 g pectin in 10 mL solvent) was used for the highest yield of pectin production and was suggested as an environmentally friendly, efficient, and time-saving emerging technology, showing superior findings over the conventional pectin extraction methods. This research group performed microwave-assisted extraction by adding pressurized water/carbon dioxide to the system with a different approach to the conventional microwave treatment in order to improve the diffusion of the solvent into the solid matrix.

Nastić et al. (Contribution 3) also introduced a novel green technology [25]. First, the bioactives rich in anthocyanin were extracted from black raspberry pomace via ultrasound-assisted extraction (UAE) with a sonotrode. The UAE system was optimized for the highest phenolic yield by investigating the extraction time and ultrasound amplitude. Afterwards, the aqueous alcoholic extract obtained under the optimal conditions was microencapsulated in glyceryl monostearate using gas-saturated solutions (PGSSs) to preserve the active material from degradation. The effects of the pressure and mass ratio of the extract to carrier were also determined. The raspberry pomace extract powder was formulated successfully, utilizing two green approaches (UAE and the supercritical CO_2_ encapsulation), to be an additive candidate in food, cosmetic, and pharmaceutical products in the future.

Romano et al. (Contribution 4) valorized the waste of the coffee sector (spent coffee grounds) as a resource for antioxidants [26]. A green approach was adopted through supercritical and liquid CO_2_ extractions. CO_2_ was modified with 5% ethanol to increase the local density around the molecules and to enhance the interaction between the solute and the solvent system. The findings of the proposed method were much better in comparison to the conventional Soxhlet extraction method with hexane and ethanol, considering the shorter extraction time (5 h versus 1 h). The proposed method was recommended for developing the coffee extract formulation and valorizing it as a fat additive, as an active packaging ingredient for the prevention of lipid oxidation, and as a flavoring ingredient in food products. 

In recent years, green technologies have gained importance regarding their use for the recovery of various bioactives from food processing waste by-products [27,28]. In this context, methods such as microwave-assisted extraction, ultrasound-assisted extraction, and supercritical extraction (mentioned above) are defined as green extraction methods [29,30,31]. However, during the preservation of aqueous extracts, the active substance is protected by trapping it in a wall material. This can be achieved by using methods such as microencapsulation to prevent degradation, as the active substances in aqueous extracts are sensitive to factors such as heat, light, and oxygen. Toprakci et al. (Contribution 5) examined the microencapsulation of extracts rich in anthocyanins, which display great health benefits but are heat-sensitive [32]. For this reason, they used the ionic gelation system, which is a simple and practical encapsulation system that does not require high temperatures. The researchers followed a completely green approach. Automatic solvent extraction, which is an environmentally friendly extraction system, was used by choosing a green solvent (aqueous alcohol, 80%). The extract rich in anthocyanins was subjected to ionic gelation to load the actives in alginate beads. The optimization of the ionic gelation system and the identification of the effects of process parameters (gelling medium concentration, alginate concentration, and time) were carried out by employing a Box–Behnken design of the RSM. The quality parameters (encapsulation efficiency and antioxidant activity), physicochemical measurements (moisture content, water activity, and bulk density), and the morphological properties demonstrated that the developed products might be potential alternative natural antioxidants for food formulations. 

To conclude, this Special Issue has contributed to some of the most popular topics of recent times, such as biorefinery, circular economy, sustainability, and life cycle assessment. These results are important as they form the basis of understanding this field for future studies. Although the relevant active substances have demonstrated their biologically active properties through in vitro studies, these studies are not sufficient to prove that these products are reliable. For this purpose, in spite of whether the proportions of active ingredients used will create a toxic effect or what effect they will have in combination with other components, their release behavior in in vitro and in vivo gastrointestinal systems and their release in relevant products should be examined comprehensively. Lastly, these findings can be utilized as preliminary information before moving on to in vivo and clinical processes.
